# Suppression of the senescence-associated secretory phenotype (SASP) in human fibroblasts using small molecule inhibitors of p38 MAP kinase and MK2

**DOI:** 10.1007/s10522-015-9610-z

**Published:** 2015-09-23

**Authors:** Dauren Alimbetov, Terence Davis, Amy J. C. Brook, Lynne S. Cox, Richard G. A. Faragher, Talgat Nurgozhin, Zhaxybay Zhumadilov, David Kipling

**Affiliations:** National Laboratory Astana, Centre for Life Sciences, Nazarbayev University, Astana, Kazakhstan; School of Medicine, Institute of Cancer and Genetics, Cardiff University, Heath Park, Cardiff, CF14 4XN UK; Department of Biochemistry, University of Oxford, South Parks Road, Oxford, OX1 3QU UK; School of Pharmacy and Biomolecular Sciences, University of Brighton, Cockcroft Building, Lewes Road, Moulsecoomb, Brighton, BN2 4GJ UK

**Keywords:** Cellular senescence, Human fibroblasts, Human ageing, SB203580, MK2.III, BIRB 796

## Abstract

Senescent cells show an altered secretome profile termed the senescence-associated secretory phenotype (SASP). There is an increasing body of evidence that suggests that the accumulation of SASP-positive senescent cells in humans is partially causal in the observed shift to a low-level pro-inflammatory state in aged individuals. This in turn suggests the SASP as a possible therapeutic target to ameliorate inflammatory conditions in the elderly, and thus a better understanding of the signalling pathways underlying the SASP are required. Prior studies using the early generation p38 MAPK inhibitor SB203580 indicated that p38 signalling was required for the SASP. In this study, we extend these observations using two next-generation p38 inhibitors (UR-13756 and BIRB 796) that have markedly improved selectivity and specificity compared to SB203580, to strengthen the evidence that the SASP is p38-dependent in human fibroblasts. BIRB 796 has an efficacy and toxicity profile that has allowed it to reach Phase III clinical trials, suggesting its possible use to suppress the SASP in vivo. We also demonstrate for the first time a requirement for signalling through the p38 downstream MK2 kinase in the regulation of the SASP using two MK2 inhibitors. Finally, we demonstrate that a commercially-available multiplex cytokine assay technology can be used to detect SASP components in the conditioned medium of cultured fibroblasts from both young and elderly donors. This assay is a high-throughput, multiplex microtitre-based assay system that is highly sensitive, with very low sample requirements, allowing it to be used for low-volume human biological fluids. Our initial studies using existing multiplex plates form the basis for a “SASP signature” assay that could be used as a high-throughput system in a clinical study setting. Our findings therefore provide important steps towards the study of, and intervention in, the SASP in human ageing and age-related disease.

**Electronic supplementary material:**

The online version of this article (doi:10.1007/s10522-015-9610-z) contains supplementary material, which is available to authorized users.

## Introduction

Cellular senescence is defined as a limited and finite ability of human cells to divide, and becomes evident through phenotypic changes in morphology, gene expression, and function (Campisi 1996; Krtolica and Campisi 2002). Essentially permanent cell-cycle arrest is the most obvious characteristic of cell senescence, whereby expression of cell-cycle-inhibitors prevents DNA replication or cell cycle progression (Herbig et al. 2004; Serrano et al. 1997). Senescence is divided into two broad categories, replicative and stress-induced senescence, although this is an operational definition describing how cells arrive at senescence; irrespective of inducer, senescent cells are typically very similar in their properties and behaviour (Toussaint et al. 2002).

Replicative senescence results from the progressive shortening of telomeres as cells divide throughout life (Bodnar et al. 1998). When the telomeres become critically short they trigger a DNA damage response involving activation of p53, which in turn up-regulates the cell-cycle-inhibitor p21^CDKN1^ resulting in cell cycle arrest and entry into senescence (Vaziri and Benchimol 1996). Several experimental manipulations such as hyperoxia, hypermitogenic signalling or oncogene expression can also trigger entry into a senescence-like state (Blagosklonny 2003; Saretzki and Von Zglinicki 2002; Shay and Roninson 2004). These interventions collectively result in the activation of the stress-induced p38 mitogen-activated protein kinase (MAPK) through a process known as stress-induced premature senescence or SIPS (Haq et al. 2002; Iwasa et al. 2003; Wang et al. 2002; Toussaint et al. 2000). The p38 MAPK can mediate cell cycle arrest directly by activating both p53 and p21^CDKN1^ (Bulavin et al. 1999; Haq et al. 2002), or indirectly via the activation of p16^INK4A^ (Deng et al. 2004; Wang et al. 2002). Because telomere dysfunction is also a stressor that results in activation of p38 (Iwasa et al. 2003), and p38 can activate p53, the two pathways may act synergistically. This places p38 at a pivotal position in the induction of cellular senescence.

Cellular senescence confers an important tumour-suppressive function (Prieur and Peeper 2008; Krtolica and Campisi 2002). However, the gradual accumulation over the life-course of senescent cells with an altered phenotype also is likely to contribute to age-related diseases and degeneration (Ovadya and Krizhanovsky 2014; Burton 2009). This is believed to be due, at least in part, to the development of what has been termed the senescence-associated secretory phenotype or SASP Coppe et al. 2008, 2010, involving a marked change in gene expression in senescent cells with increased expression of pro-inflammatory cytokines (notably IL-6 and IL-8), chemokines, matrix metalloproteinases, and other proteins that have the potential to alter the local tissue microenvironment (Coppe et al. 2010). The SASP does not develop immediately after a senescence-inducing stimulus, but once established it can persist for long periods (Coppe et al. 2008; Rodier et al. 2009).

The SASP can potentially have both deleterious and beneficial effects, depending upon the biological context (Freund et al. 2011). Beneficial aspects appear to include a reinforcement of the anti-tumour cellular growth arrest (Acosta et al. 2008), a positive contribution to the clearance of senescent cells by the immune system (Xue et al. 2007), and suppression of fibrotic scar formation (Krizhanovsky et al. 2008). Detrimental effects include the promotion of malignant phenotypes of neighbouring cells and accelerated tumour growth (Coppe et al. 2010; Krtolica et al. 2001; Gonzalez et al. 2015). Moreover, many SASP factors are pro-inflammatory, thus the increased accumulation of senescent cells with age (Herbig et al. 2006; Jeyapalan et al. 2007) may contribute to the low-level chronic inflammation that is a hallmark of aged mammalian tissues, and provides one route whereby senescent cells can have deleterious effects in the context of ageing (Brassard et al. 2015; Davis and Kipling 2006; Freund et al. 2011; Rodier and Campisi 2011). Given the proposed importance of the SASP, an understanding of its regulation is crucial, particularly in the context of developing potential future therapies for ageing processes.

p38 MAPK is necessary and sufficient for the development of a SASP in cells induced to senesce by direct X-ray induced DNA damage or by expression of oncogenic ras (Freund et al. 2011). Since many features of senescence result from p38 activation (Iwasa et al. 2003), the p38 axis is an attractive target for therapeutic intervention in aspects of human ageing and age-related degeneration and diseases that are postulated to be connected to senescence at the cellular level. In practice, however, p38 MAPK has not been a straightforward target for drug development. The most commonly used inhibitor SB203580 is unsuitable for in vivo use due to specificity and toxicity issues (Goldstein et al. 2010). Many p38 inhibitors with better inhibitory properties than SB203580 (increased potency, better target specificity) have been used in clinical trials (Force et al. 2004), but their long-term use has revealed numerous problems, including examples both of lack of efficacy, and mild liver and CNS toxicity (Goldstein et al. 2010). As such, none have yet been developed into full therapeutic use.

These difficulties associated with p38 as a drug target suggest that other approaches should be explored in parallel. A major target of p38 is the kinase MK2, which is involved in the induction of the stress fibres that are characteristic of senescent cells via phosphorylation of HSP27 (Guay et al. 1997), cell cycle arrest via phosphorylation of the phosphatases CDC25B/C leading to the inhibition of cyclin/CDK complexes (Manke et al. 2005; Kumar et al. 2003), and the phosphorylation and inactivation of tristetraprolin (TTP) which results in the stabilisation of mRNAs encoding many pro-inflammatory factors (Neininger et al. 2002; Ross et al. 2012; Hitti et al. 2006). These lines of evidence point to MK2 being a major kinase involved in cellular senescent phenotypes including, possibly, the SASP, although the latter has not been established. This is supported by evidence showing that TTP knockout mice are hyper-inflammatory (Taylor et al. 1996), whereas MK2 knockout mice are inflammation-resistant (Hegen et al. 2006). It is interesting to note that MK2 knockout mice are viable, whereas p38 knockout mice do not survive (Ronkina et al. 2008). This indicates that MK2 is not essential for organismal survival, which in turn gives hope that chemical inhibition of MK2 may be less problematic than inhibition of p38 (Duraisamy et al. 2008).

In this current study, we explore the expression of the SASP in several strains of human fibroblasts from individuals of varying ages, and examine the effects of a range of p38 and MK2 inhibitors to further elucidate the molecular pathways involved in SASP expression. We find that cells from both young and old donors have low background inflammatory profiles but demonstrate a robust SASP as they approach senescence. We further show that inhibition of either p38 MAPK or MK2 can suppress the SASP.

## Materials and methods

### Cells and cell growth

Four strains of human primary normal dermal fibroblasts (NDFs) were obtained from the Coriell Cell Repositories (Camden, NJ, USA) from a range of elderly and younger individuals with a range of replicative capabilities (Cristofalo et al. 1998): AG16409 (donor age 12), AG07719A (donor age 28), AG08433 (donor age 94) and AG11081 (donor age 79). Cells were cultured at 37 °C in a humidified incubator with 3 % O_2_ and 5 % CO_2_, and all medium was pre-conditioned in the 3 % O_2_ environment for 1 h prior to use.

For long-term proliferation to replicative senescence, cells were grown in DMEM supplemented with 10 % FCS as previously described (Davis and Kipling 2009). Population doublings (PDs) were calculated according to the formula: PD = log(*N*_t_/*N*_o_)/log(2), where *N*_t_ is number of cells harvested and *N*_o_ is the initial number of cells seeded.

### Protein kinase inhibitors

The protein kinase inhibitor SB203580 was obtained from Tocris Chemical Co. (Bristol, UK). The kinase inhibitors BIRB 796 and UR-13756 were synthesised according to (Bagley et al. 2006, 2010). The MK2 inhibitors MK2.III (Anderson et al. 2007) and PF-3644022 (Mourey et al. 2010) were obtained from Merck (UK) and Tocris Chemical Co. (Bristol, UK) respectively.

### Conditioned medium collection and SASP assay

Conditioned medium for detecting SASP components was collected according to (Coppe et al. 2008) with slight modifications. In brief, 10,000–30,000 proliferating cells, or 30,000–50,000 senescent cells were seeded into 6 well dishes in DMEM containing 10 % FCS and cultured at 37 °C for 4 days. The medium was then replaced with DMEM containing 0.5 % FCS and the cells grown for a further 48 h at 37 °C. Finally the medium was replaced with DMEM without FCS and the cells incubated for 24 h. The resultant conditioned medium (3 ml) was harvested, snap-frozen as 0.5 ml aliquots in liquid nitrogen and stored at −80 °C prior to analysis. Final cell numbers were determined for all samples and analyte values normalised against cell number. This method is the standard for analyzing SASP components as used in the literature (Coppe et al. 2008).

The meso scale discovery (MSD) multiplex immunoassay (K15007B-1) was used for the simultaneous detection of SASP components GM-CSF, IL-1β, Il-6, IL-8, IL-10, TNFα and IFNγ in conditioned medium using a Sector Imager SI-6000 according to the manufacturer’s instructions. MIP-3α (Κ151ΒΕΒ-1) detection used an MSD singleplex assay. For ELISA-based detection of IL-6 in conditioned medium, the 96-well microplate Quantikine IL-6 kit (#D6050 from R&D Systems) was used according to the manufacturer’s instructions.

For assessing the effects of the various kinase inhibitors on the SASP the culture medium was supplemented with the inhibitor dissolved in DMSO, with the inhibitors being present throughout the experiment and replaced at each medium change. The inhibitors used were BIRB 796 (2.5 µM), SB203580 (2.5 or 10.0 µM), UR-13756 (2.5 µM), MK2.III (1.0, 2.5 and 5.0 µM) and PF-3644022 (1.0, 2.5 and 5.0 µM): all concentrations given are the final concentration in the growth medium. For controls an equivalent volume of the solvent DMSO was added to the medium at a final concentration of 0.1 % (v/v). The p38 inhibitors fully inhibit anisomycin-induced p38/MK2-dependent phosphorylation of HSP27 at these concentrations (Bagley et al. 2006, 2010; Davis et al. 2005). The MK2 inhibitors are fully inhibitory for MK2-dependent HSP27 phosphorylation above 2.0 µM (Davis et al. 2013; Bagley et al. 2015).

### Detection of senescence-associated β-galactosidase activity

Endogenous mammalian senescence-associated β-galactosidase activity (SA-β-gal) was assessed histochemically as described previously (Davis et al. 2003). The proportion of β-galactosidase positive cells was assessed in a total count of 500 cells per sample.

### BrdU incorporation assays

Cells were seeded into 6-well dishes cultured for 48 h in DMEM containing 10 % FCS, and then incubated in the presence of bromodeoxyuridine (BrdU) at 10 µM for 2 h. After incubation cells were processed prior to BrdU detection as described (Davis et al. 2005). BrdU incorporation was detected by overnight incubation with a monoclonal rat anti-BrdU antibody labelled with fluorescein isothiocyanate (Abcam, ab74545) with DNA counterstained with DAPI. Images were captured using fluorescent microscopy using a Zeiss Axiovert 135 M microscope, and the BrdU labelling index was calculated as (labelled cells/total cells) × 100 %.

## Results

### Detection of the SASP in replicatively senescent cells

**Fig. 1:**
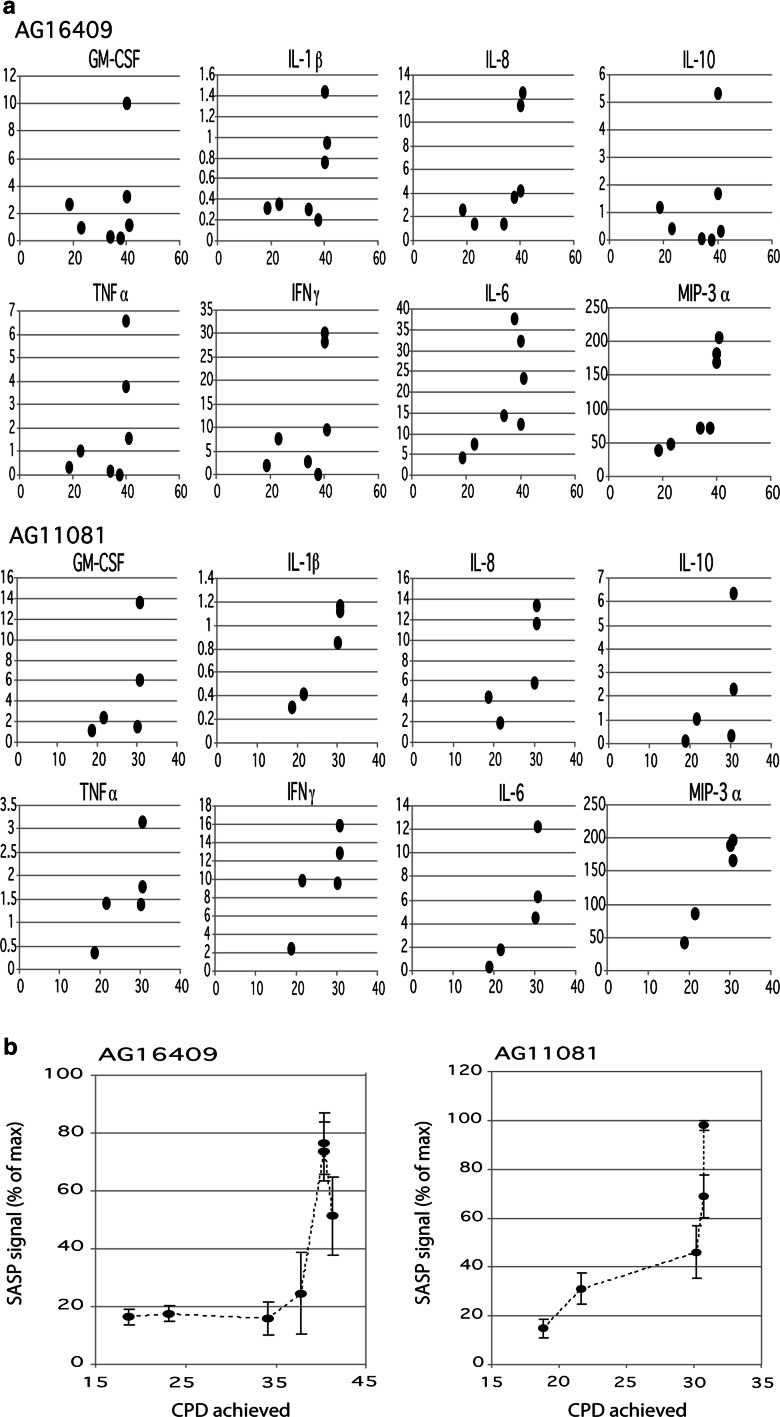
Cytokine levels versus cumulative population doublings (CPD) for AG16409 and AG11081 cells. **a** Plot of cytokine levels versus CPD for cell strains AG16409 (*top panels*) and AG11081 (*middle panels*) and each analyte. Data is plotted as pg analyte/ml conditioned medium (normalised to 30,000 cells) versus CPD achieved. **b** The data from a is calculated for each PD point as a percentage of the maximum level seen in the senescent cells for each analyte. Then the average is calculated for all eight cytokines and plotted against the CPD. The data is shown as the average % ± the SE of the means of each individual analyte

To determine whether the SASP is related to donor age, and whether it can be equally detected in senescent cells from young and old donors, we analysed a panel of 8 proinflammatory cytokines in conditioned medium from proliferating cells at early population doublings in culture, and as those cells reached senescence (as indicated by a loss of BrdU labelling, accumulation of SA-β-gal staining and cessation of proliferation—see Table S1). Fibroblast strains AG16409 (donor age 12) and AG11081 (donor age 79) were initially studied. SASP components were measured quantitatively using MSD immunoassays. Cytokine levels detected were normalised to cell number and then expressed as pg/ml medium per 30,000 cells. The levels of each of the eight cytokines were plotted as a function of cumulative population doublings: the cytokine levels remained low until the cells were very close to, or at, replicative senescence, at which point the levels rose sharply (Fig. 1a).

The data for each cytokine at each PD point were calculated as a percentage of the maximal level seen at senescence and then the data were combined to give a single aggregate “SASP signature” that showed a large increase when the cells reached replicative senescence (Fig. 1b). This is seen clearly in the AG16409 fibroblast strain (young donor) as the SASP signal is low and essentially constant up to the point when the cells are only 6 PD from senescence, after which the signal increased rapidly with CPD, with the signal variability also increasing markedly. The SASP signature appeared to increase at an earlier stage in the growth curve for the aged donor AG11081 cells.

**Fig. 2:**
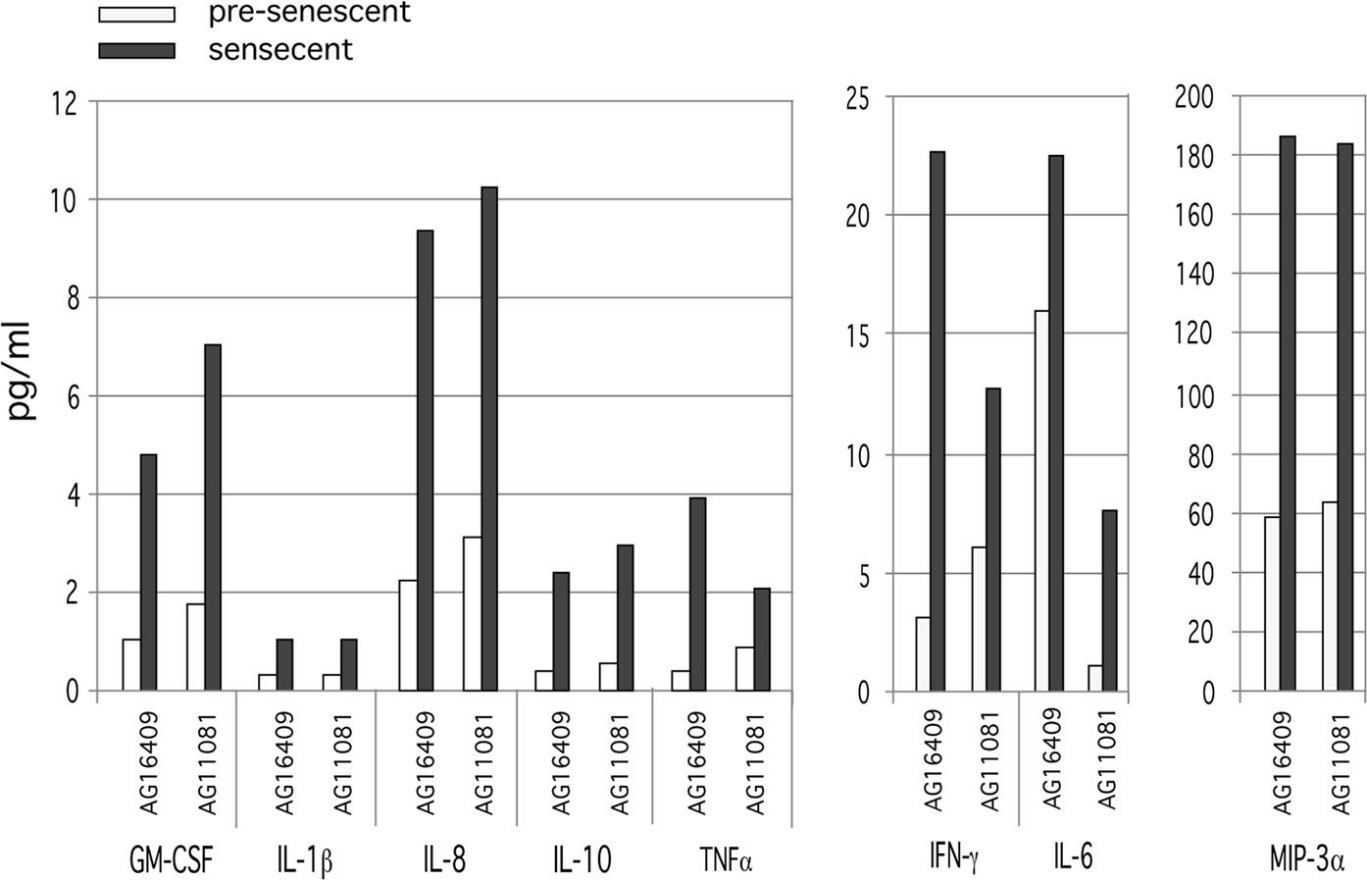
Bar chart showing cytokine levels in pg/ml medium for pre-senescent and senescent AG16409 and AG11081 cells. The protein levels are normalised to 30,000 cells (see Table S1 for cell CPD details)

To permit easier comparison of the difference between proliferating and senescent cells in terms of SASP, we then pooled data from all proliferating cells and plotted the mean against pooled SASP data from all senescent cells of the same strain (see Table S1 for pools) For both cell lines, the cytokine levels were higher in medium from the senescent cell populations compared to the medium taken from proliferating cells (Fig. 2). In addition, both the 12 year old donor (AG16409) and the 79 year old donor (AG11081) cells secreted similar patterns and concentrations of cytokines (with the possible exception of IL-6). Thus cells from both young and old donors secrete only low levels of inflammatory cytokines while they are still proliferating but both produce a robust SASP once senescent.

Although we clearly detected a SASP signal using the MSD immunoassays, the levels of many of the cytokines on the multiplex plates used were relatively low. For the remaining part of the study we therefore focused on IL-6, a canonical surrogate for the SASP, as it shows a good SASP response in an ELISA based assay.

**Fig. 3:**
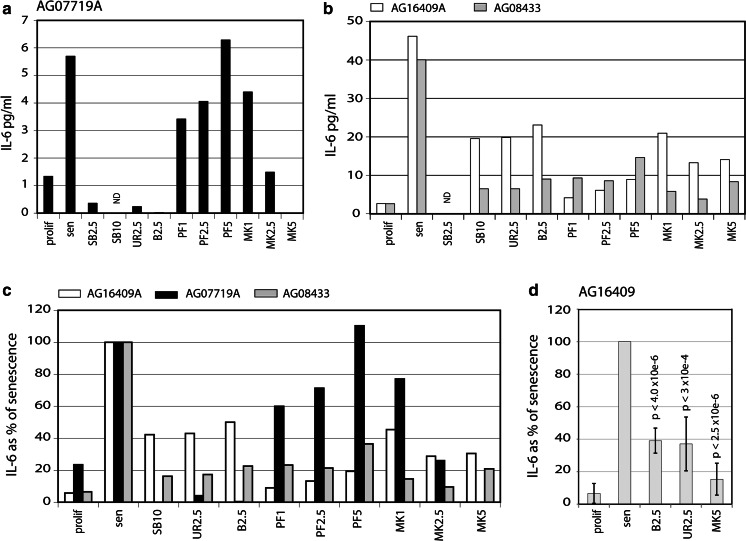
Effects of p38 and MK2 inhibitors on IL-6 expression. Levels of IL-6 in pg/ml conditioned medium (normalised to 30,000 cells) for **a** AG07719A cells, **b** AG16409 and AG08433 cells. **c** Data from **a** and **b** expressed as a percentage of IL-6 at senescence for each strain. **d** Average IL-6 levels shown as a percentage of the level seen at senescence for AG16409 cells using four biological replicates. Only one inhibitor concentration has been used. Values are mean ± SD, *p* values are compared to the levels seen at senescence; Student *t* Test. Prolif and sen refer to cells at low CPD and senescence respectively (see Table S2 for cell CPD details). The rest of the data refer to inhibitor-treated senescent cells. Key to inhibitors on *x* axes: SB = SB203580, UR = UR-13756, B = BIRB 796, PF = PF-3644022, MK = MK2.III; the numerical values are µM. ND is not determined

To determine whether SASP induction occurs in cells obtained from both young and old individuals as they undergo replicative senescence, three Normal diploid fibroblast strains from donors ranging in age from twelve to 94 years were cultured continuously until they reached replicative senescence (defined by lack of any cell proliferation over at least 30 days, a BrdU labelling index of less than 4.5 % and SA-β-gal staining of greater than 80 % (see Table S2 for details)). Proliferating (low CPD) AG16409A fibroblasts from the youngest donor expressed low levels of IL-6 (2.66 pg/ml) (Fig. 3), which increased to 46.16 pg/ml when the cells reached senescence, confirming a robust induction of the IL-6 component of the SASP. The strain from a 28 year old individual, AG07719A, had much lower IL-6 levels in proliferating cells (1.34 pg/ml) that were also elevated in senescent cells (5.7 pg/ml). Similar results, though with a much greater fold IL-6 induction, were seen for the strain AG08433 from a 94 year old individual with IL-6 levels at 2.6 pg/ml for proliferating low CPD cells, increasing to 40.04 pg/ml for senescent cells (Fig. 3b; see also Table S3). Our results clearly show that fibroblasts from old donors do not secrete high levels of IL-6 until senescence but then produce a robust SASP; this is a highly important finding as clinical use of drugs to alleviate SASP in senescence is likely to focus on elderly patients.

### Suppression of the SASP using p38 inhibitors

To test whether inhibition of the p38 MAPK signalling axis impacts on the SASP, proliferating and senescent fibroblasts were then treated with various inhibitors of p38 MAPK or MK2 (see Fig. 3; Table S3) and conditioned medium tested for IL-6 levels (normalised to the cell number and expressed as pg/ml medium per 30,000 cells). We find that inhibition of p38 MAPK reduced senescence-associated IL-6 secretion in all three cell strains (Table S3; Fig. 3), though variation between cell strains was observed e.g. in AG16409A cells IL-6 levels are only reduced to about 40 % of that seen in senescent cells (Fig. 3b, c), while for AG08433 cells IL-6 levels were reduced by more than 80 %. In addition, within each cell strain, all three p38 inhibitors tested showed similar efficacy in IL-6 suppression. Finally, as the levels of IL-6 vary considerably between cell strains and the effects of the inhibitors are also variable dependent upon strain used, we repeated the experiment using proliferating and senescent AG16409 cells using four different samples as a test of reproducibility (Fig. 3d). As can be seen, a robust SASP is seen at senescence (as measured by the increase in IL-6) that is supressed using a variety of inhibitors with very high reproducibility (as indicated by the *p* values).

### Suppression of the SASP using MK2 inhibitors

We next determined the role of MK2 in IL-6 induction at senescence. For this we used two commercially available MK2 inhibitors, PF-3644022 and MK2.III, both used at a range of concentrations. The effects of the two inhibitors were cell strain dependent (Table S3). For AG07719A cells, PF-3644022 had no effect on IL-6 levels when used at 5 µM, but reduced IL-6 levels slightly when used at 1.0 and 2.5 µM (Fig. 3a). For strain AG16409A, however, PF-3644022 treatment (at 1.0 and 2.5 µM) decreased IL-6 levels in senescent cells to almost to the level seen in young cells (Fig. 3b, c), albeit being less effective at 5.0 µM. The magnitude of the effects of PF-3644022 were smaller in AG08433, the strain from the elderly individual (Fig. 3b, c), but showed similar trends to that seen in strain AG016409A from the young donor. By contrast, MK2.III at 5 µM progressively reduced secreted IL-6 levels to essentially zero in AG07719A cells, and to about 25 % of that seen in proliferating cells for AG16409A and AG08433 cells (Fig. 3a–c). This MK2 inhibitor was therefore at least as effective as the p38 inhibitors in IL-6 suppression in all three strains (Fig. 3c). Thus IL-6 secretion by senescent cells can be suppressed by inhibition of the MK2 kinase in cells from both young and old donors.

## Discussion

There is an increasing body of evidence that suggests that the accumulation of senescent cells with age in humans is at least partially causal in the well-observed shift to a low-level pro-inflammatory state (Freund et al. 2011; Rodier and Campisi 2011; Burton 2009). Although definitive proof of the source of these cytokines has yet to be obtained, there is certainly a plausible argument for them making a contribution to age-related diseases and degenerations, many of which have inflammation as known risk factors. To explore this further and potentially exploit these findings in a translational setting requires methodology suitable for the detection of SASP analytes in human clinical material, potentially as part of a large cohort study, together with drugs suitable for human clinical trials. This would then open the way to considering potential human clinical trials of short-term effect of drug inhibition of the SASP by measuring circulating pro-inflammatory cytokines in the elderly (or in human premature ageing syndromes such as Werner Syndrome, which are associated with a marked activation of the SASP-regulating p38 MAPK pathway), and then follow-on work to assess the physiological and clinical outcomes (Davis and Kipling 2006).

In the first part of this current study (Fig. 1) we demonstrate that commercially available multiplex cytokine assay technology (MSD) can be used to detect SASP components in the conditioned medium of cultured fibroblasts from both young and elderly donors. We note that many laboratory studies on the SASP use fibroblast strains from young donors (Freund et al. 2011; Coppe et al. 2008, 2010). Whilst these have a long replicative lifespan and therefore produce a very clear and experimentally tractable distinction between non-senescent (proliferating) and senescent (arrested) cells, and a very clear SASP as a result, we are cognisant that in vivo any human clinical studies would need to measure SASP generated from cells in an elderly person. It is therefore important that our data demonstrate that a SASP is generated by senescent cells from elderly donors, and that it is a consequence of cellular senescence not donor age. Our findings therefore add the novel finding of robust SASP responses in senescent cells from elderly individuals to the many reports in the literature that typically use cells derived from young (often new-born) individuals.

The MSD platform is a high-throughput, multiplex microtitre-based assay system. It is designed with a view to facilitating large-scale population-based human studies. It is very sensitive, with very low sample requirements, which enables it to be used for low-volume human biological fluids. Our initial studies using existing multiplex MSD plates (Fig. 1a) are encouraging that this would form the basis for a “SASP signature” assay (Fig. 1b) that could be used in a clinical study setting. Further work is now needed to refine the choice of analytes on the MSD multiplex to give an optimum SASP detection system.

Prior studies had indicated a role for p38 MAPK in expression of the SASP (Freund et al. 2011), although the work was limited to the use of the p38 inhibitor SB203580 that is known to be confounded by significant off-target effects (Goldstein et al. 2010). To strengthen the evidence for a role of p38 MAPK signalling in regulating the SASP we used two next-generation p38 inhibitors (UR-13756 and BIRB 796) that have markedly improved selectivity and specificity compared to SB203580 (Bain et al. 2007; Mihara et al. 2008). As shown in Fig. 3, both compounds are highly effective at blocking the SASP in senescent cells when used at concentrations that are known to inhibit p38 MAPK. These data therefore provide further evidence that signalling through p38 MAPK is required for the SASP in human cells. As an aside, we note that BIRB 796 has a efficacy and toxicity profile that has allowed it to reach Phase III clinical trials for inflammatory conditions such as Crohn’s disease, rheumatoid arthritis, ulcerative colitis and psoriasis (Force et al. 2004), which demonstrates the progress that has been made in bringing SASP-suppressive drugs into human clinical use.

There are well-documented problems with the development of drugs that both target p38 MAPK and are safe and effective for long-term clinical use (Goldstein et al. 2010). The repeated issues of toxicity that have been seen with different p38 inhibitors have raised the possibility that some toxicity may be an inevitable effect of inhibiting p38 itself. This idea gains additional strength when we consider the lethality of p38 knockout in the mouse, as compared to the viability of knockouts of the downstream target effector kinase MK2 (Ronkina et al. 2008). Such considerations have led to efforts to target MK2, in the hope that by inhibiting a reduced number of pathways and targets (compared to the upstream p38 MAPK) this could generate potential drugs with a better toxicity profile (Duraisamy et al. 2008). Work on MK2 inhibitors has, however, faced problems due to the relatively shallow ATP pocket on the protein, which has presented a challenge to the synthesis of high specificity MK2 inhibitors (Schlapbach and Huppertz 2009). The new generation of MK2 inhibitors are exemplified by PF-3644022 and MK2.III. In our experience (data not shown) PF can demonstrate adverse effects on cell viability and phenotype at concentrations not substantially greater than required for full inhibition of MK2, and it would thus seem likely that at lower concentrations PF-3644022 will nevertheless cause some degree of stress signalling. MK2.III in contrast shows a much better toxicity profile, with few obvious adverse effects on cultured fibroblasts at concentrations sufficient to maximally inhibit MK2 (data not shown). We therefore tested these two MK2 inhibitors in parallel to our panel of p38 inhibitors (SB203580, UR-13756 and BIRB 796) for inhibition of the SASP. PF-3644022 does result in a degree of SASP suppression, but this effect is rapidly lost at higher concentrations (5 µM) where in fact levels of secreted IL-6 can exceed those seen in untreated senescent cells (Fig. 3). Given our observations of significant adverse pleiotropic effects of PF at 5 µM it seems plausible that the data for this compound is a mixture of inhibition of the SASP at low levels, which is then swamped by non-specific MK2-independent stress-induced IL-6 induction at higher PF levels. In contrast, MK2.III has a much better toxicity profile on cultured cells, and shows initial inhibition of the SASP at 1 µM (which is less than the level needed for full MK2 inhibition), progressing to higher levels of inhibition with almost 100 % inhibition at 5 µM, at which concentration MK2.III shows little cell toxicity (Davis et al. 2013). These data provide support for the continuing development of MK2 inhibitors for potential in vivo use for inflammatory conditions, and perhaps even for human ageing (Duraisamy et al. 2008).

In summary, this study demonstrates that a SASP can be detected in senescent cells derived from elderly donors and that a “SASP signature” multiplex assay can be implemented on a high-throughput system that could potentially be used for human clinical and population studies. We provide further evidence by the use of improved p38 inhibitors that the p38/MK2 axis is critical in regulating the human SASP. Finally, for the first time we demonstrate an essential role for the downstream MK2 kinase in regulation of the SASP. These data provide important steps towards the study and intervention in the SASP in human ageing and age-related disease.

## Electronic supplementary material

Below is the link to the electronic supplementary material.

Supplementary material 1 (DOCX 69 kb)

Supplementary material 2 (DOCX 59 kb)
