# Supplementary material for: Suppression of the senescence-associated secretory phenotype (SASP) in human fibroblasts using small molecule inhibitors of p38 MAP kinase and MK2
Source: Biogerontology. 2015 Sep 23;17:305–15. doi: 10.1007/s10522-015-9610-z (PMC4819486; doi:10.1007/s10522-015-9610-z)
Supplement: Supplementary file 1 — Supplementary material 1 (DOCX 69 kb) [file 10522_2015_9610_MOESM1_ESM.docx]

Alimbetov et al., (2015) Suppression of the senescence-associated secretory phenotype (SASP) in human fibroblasts using small molecule inhibitors of p38 and MK2. Biogerontology.

**Table S1. Cell strain information for the Meso Scale Diagnostics SASP assays**

| Strain ID | Donor Information | | | Pre-senescent Pools | | | Senescent Pools | | |
| --- | --- | --- | --- | --- | --- | --- | --- | --- | --- |
|  | Age  [Years] | Gender | Ethnicity | PD  (% of rep capacity)^a^ | SAβGal [% +ve cells] | BrdU Index [% +ve cells] | PD  (% of rep capacity)^a^ | SAβGal [% +ve cells] | BrdU Index [% +ve cells] |
| AG16409 | 12 | Male | Caucasian | 18.6 (45.4) | 9.2 | 22.0 | 40.1 (97.8) | 100.0 | 3.0 |
|  |  |  |  | 23.0 (56.1) | 4.2 | 31.0 | 40.1 (97.8) | 100.0 | 5.0 |
|  |  |  |  | 34.0 (82.9) | 22.0 | 13.0 | 41.0 (100) | 100.0 | 0.0 |
|  |  |  |  | 37.7 (91.9) | 24.0 | 12.0 |  |  |  |
| AG11081 | 79 | Female | Caucasian | 18.8 (61.3) | 6.6 | 5.0 | 30.7 (100) | 100.0 | 0.0 |
|  |  |  |  | 21.6 (70.4) | 6.2 | 10.0 | 30.7 (100) | 93.6 | 0.0 |
|  |  |  |  |  |  |  | 30.2 (98.4) | 98.2 | 1.0 |

^a^ % of replicative capacity defined as PD achieved/maximum PD at senescence.

**Table S2. Cell strain information for IL-6 ELISA assays**

| Strain ID | Donor Information | | | Initial PD  (% of rep capacity)^a^ | Predicted Lifespan [CPD]^b^ | PD  Obtained  (% of rep capacity)^a^ | Young Cell Phenotype | | Senescent Cell Phenotype | |
| --- | --- | --- | --- | --- | --- | --- | --- | --- | --- | --- |
|  | Age [Years] | Gender | Ethnicity |  |  |  | SAβGal  [% +ve cells]  @ day 36 | BrdU Index  [% +ve cells]  @ day 36 | SAβGal  [% +ve cells] endpoint | BrdU Index  [% +ve cells] endpoint |
| AG16409A^c^ | 12 | Male | Caucasian | 10.1 (17.9) | 50.0 | 56.3 (100) | 2.2 | 51.6 | 82.9 | 2.5 |
| AG07719A | 28 | Female | Caucasian | 16.0 (42.7) | 34-37 | 37.5 (100) | 0.4 | 25.6 | 87.3 | 2.0 |
| AG08433 | 94 | Male | Caucasian | 15.5 (44.2) | 34.0 | 35.1 (100) | 9.2 | 19.0 | 88.2 | 4.4 |

^a^ % of replicative capacity defined as PD achieved/maximum PD at senescence.

^b^ Predicted lifespan in cumulative PDs (CPD) (Cristofalo et al. 1998).

^c^ note that the AG16409A cells used here are a different batch to the AG16409 cells listed in Table S1. Different batches of the same cell strain are known to

sometimes have different replicative capacities (Cristofalo et al. 1998).
