# Supplementary material for: Suppression of the senescence-associated secretory phenotype (SASP) in human fibroblasts using small molecule inhibitors of p38 MAP kinase and MK2
Source: Biogerontology. 2015 Sep 23;17:305–15. doi: 10.1007/s10522-015-9610-z (PMC4819486; doi:10.1007/s10522-015-9610-z)
Supplement: Supplementary file 2 — Supplementary material 2 (DOCX 59 kb) [file 10522_2015_9610_MOESM2_ESM.docx]

Alimbetov et al., (2015) Suppression of the senescence-associated secretory phenotype (SASP) in human fibroblasts using small molecule inhibitors of p38 and MK2. Biogerontology.

**Table S3. IL-6 levels in inhibitor treated cells**

IL-6 levels (pg/ml) for Fig. 3.

strain AG16409A AG07719A AG08433

condition^a^ Inhibitor

target

Proliferating - 2.66 1.34 2.60

senescent - 46.16 5.70 40.04

SB2.5 p38 nd^b^ 0.36 nd^b^

SB10 p38 19.53 nd^b^ 6.50

UR2.5 p38 19.84 0.24 6.93

B2.5 p38 23.09 0.02 9.04

PF1 MK2 4.16 3.42 9.31

PF2.5 MK2 6.12 4.06 8.57

PF5 MK2 8.92 6.29 14.61

MK1 MK2 20.97 4.40 5.84

MK2.5 MK2 13.27 1.49 3.84

MK5 MK2 14.09 0.00 8.35

^a^ For definitions see legend to Fig. 3.

^b^ nd = not done
